# Bacterial and fungal profile, drug resistance pattern and associated factors of isolates recovered from blood samples of patients referred to Ethiopian Public Health Institute: cross-sectional study

**DOI:** 10.1186/s12879-021-06896-w

**Published:** 2021-11-29

**Authors:** Etsehiwot Adamu Tsegaye, Dejenie Shiferaw Teklu, Zelalem Tazu Bonger, Abebe Aseffa Negeri, Tesfaye Legesse Bedada, Adane Bitew

**Affiliations:** 1 National Clinical Bacteriology and Mycology Case Team, Ethiopian Public Health Institute, Addis Ababa, Ethiopia; 2 Department of Medical Laboratory Science, College of Health Science, Addis Ababa University, Addis Ababa, Ethiopia; 3 Departement of Statistics, College of Natural and computational science, Addis Ababa University, Addis Ababa, Ethiopia

**Keywords:** Blood stream infections, Bacterial and fungal profile, Antimicrobial resistance pattern, Risk factor, Ethiopia

## Abstract

**Background:**

Blood stream infections are serious infections that usually induce prolongation of hospital stay, morbidity and mortality in several countries including Ethiopia. The aim of this study was to determine bacterial and fungal profile, their drug resistance patterns, and risk factors associated with blood stream infections.

**Methods:**

A cross sectional study design was conducted from February 23 to June 23, 2020 at Ethiopian public health. A structured questionnaire was used to collect data on socio-demographic factors and clinical conditions. Blood specimens were analyzed using standard microbiological techniques. Antimicrobial susceptibility tests were performed using Kirby–Bauer disc diffusion technique and Vitek compact 2. Simple and multiple logistic regressions were used to assess the potential risk factors.

**Results:**

A total of 175 pathogens isolated from 346 blood specimens. Of these, 60% Gram-negative bacteria, 30.86% Gram-positive bacteria and 9.14% fungal isolates were identified. *Burkholderia cepacia* and *Coagulase negative staphylococcus* were the predominant pathogen among Gram-negative and Gram-positive bacteria respectively. Among fungus, *Candida krusei* (56.25%) was the most predominant isolate. The highest proportions of antibacterial resistance were observed among 3rd generation cephalosporin and penicillin. Most fungal isolates expressed resistance to fluconazole. Sex (P = 0.007), age (P < 0.001) and use of invasive medical devices (P = 0.003) were identified as risk factors for bacterial blood stream infections.

**Conclusion:**

The study showed high prevalence of blood stream infection was due to *B. cepacia* and non-*C. albicans* spp. This finding alarming ongoing investigation of blood stream infection is important for recognizing future potential preventive strategies including environmental hygiene and management of comorbid medical diseases to reduce the problem.

## Background

Blood stream infections (BSIs) are the major public health problems that are responsible for the cause of self-limited infection to death worldwide [1]. These infections can be intravascular or extravascular. Most bacterial species and many fungal species have been associated with extravascular bloodstream infections [2].

According to the global report of the World Health Organization, sepsis affected 49 million individuals and related to approximately 11 million potentially unavoidable deaths worldwide [3]. In the United States, there are about 200,000 cases of bacteria and fungi occurred annually, with mortality rates ranging from 20 to 50% [4].

Several recent studies have shown that the conditions that predispose an individual to BSIs vary with age, gender, and underlying diseases. More than half of all cases of global sepsis occurred among adolescents and children [5]. There is around 53% mortality rate among children in sub-Saharan Africa, including Ethiopia [6]. The elderly population is exposed to BSIs associated with the use of a variety of medical procedures [7].

Diabetes mellitus, renal failure requiring dialysis, widespread use of broad-spectrum antibiotics, hepatic cirrhosis, gender, age, and malignancy are the main risk factors that lead to BSIs [8, 9]. Other factors that enhance BSIs among hospitalized patients include decreased immunity, increased variety of medical procedures and the transmission of drug-resistant bacteria among crowded hospital populations [10].

As many studies highlighted, bacteria are the primary causative agents of BSIs [11–13]. However, fungal pathogens began to be recognized as important causes of disease related to increasing numbers of immunocompromised patients and nosocomial infections. Among them, 65% of the BSIs cases are induced by Candida which is responsible for raising the number of mortality rate by 38% and extending hospital stays of 30 days [14].

A large variety of organisms can cause bloodstream infections, which vary by institution and geographic distribution [15]. Coagulase-negative staphylococcus, *Staphylococcus aureus* and *Enterococcus* spp. are the most common BSIs causing Gram-positive bacterial pathogens. *Klebsiella pneumoniae*, *Escherichia coli*, *Pseudomonas* spp. and *Acinetobacter baumannii* are also the commonest among Gram-negative organisms. *Candida glabrata*, *C. parapsilosis*, *C. tropicalis*, and *C. krusei* also become frequent from fungal species [16].

In Ethiopia, systemic review done in bloodstream infections showed that the proportion of blood stream infections ranged from (12.84–18.15%). The common pathogens isolated from bacterial infected patients were *S. aureus* and *E. coli* [17]. However, it is difficult to generalize these findings to all levels of health care facilities in Ethiopia.

Blood culture remains the gold standard for bacterial and fungal detection from BSIs. Irrational use of drugs has led to an increase of multidrug-resistant organisms and thus worsened the condition. Therefore, preliminary results of Gram staining, final identification and AST results are essential for providing safe, effective, and efficient care of patients [16, 18].

The burden of blood streams infections due to antifungal resistance and associated risk factors in Ethiopia remains poorly reported. Although previous studies have been performed in the same laboratory, they have been limited to bacterial causative agents and showed the past trends of the pathogen [19]. Therefore, this study was designed to determine the current status of organisms causing bloodstream infections, their antimicrobial-resistant patterns and associated factors related to the infections among patients referred to Ethiopian Public Health, National clinical bacteriology, and mycology reference laboratory.

## Methods

### Study design, period and setting

An institutional based cross-sectional study was conducted on blood specimens from February 23 to June 23, 2020. All methods were performed in accordance with the relevant guidelines and regulations. The specimens were referred from all health care facilities in the chain of referral systems of the Department of Clinical Bacteriology and Mycology laboratory. The laboratory is the national referral laboratory in the country responsible for providing high-level diagnostic laboratory testing services for patients and specimens referred from all regional and federal health facilities. It was accredited by the Ethiopian National Accreditation office. In addition, the laboratory conducts research, capacity of regional and federal laboratory and participates in AMR surveillance. The health care facilities involved during the study period were Ras desta Hospital, Abet Hospital, St Peter Hospital, Federal Police Hospital, Yekatit Hospital, Minilik Hospital, Ring Road Hospital, Girum Hospital and Armed Force Hospital.

### Sample collection and processing

Aseptic precautions were followed to collect a total of 346 blood samples from suspected patients. Blood culture bottle was prepared with 50 ml and 25 ml tryptic soy broth containing 0.025% of sodium polyanethol sulphonate (SPS) as anticoagulant for adult and for children respectively. Two bottle of blood culture were used with 10 ml volume for adult, 5 ml for children and 2 ml for neonates.

A blind subculture was done after overnight incubation to appropriate solid culture media. Bottles that showed turbidity was subjected to Gram staining followed by sub-cultured onto blood agar plates (Oxoid Ltd, UK), chocolate agar plates (Oxoid Ltd, UK) and MacConkey agar plates (Oxoid Ltd, UK). Blood agar and chocolate agar plates were incubated in 5% CO_2_ for 24–72 h. MacConkey agar plates were placed aerobically for 24 h. Two plates of sabouraud dextrose agar plates were inoculated and incubated at 37 °C and 25 °C. Identification of bacterial isolates was carried out according to the standard bacteriological technique. A terminal sub culturing was done on chocolate agar for bottles that did not show visible growth within 7 days before being reported as negative. Species identification of some bacteria and yeasts were determined by the automated Vitek 2 compact machine (bioMérieux, France) using GN and YS 07 cards respectively.

### Antibiotic susceptibility testing

Antibiotic susceptibility testing was conducted on Muller-Hinton agar by Kirby–Bauer Disk diffusion method according to Clinical Laboratory Standard Institute (CLSI) guidelines 2021 [20]. Antimicrobial agents used for testing bacterial isolates were Penicillin (MIC), oxacillin (30 µg), gentamycin (10 µg), vancomycin (MIC), vancomycin (30 µg), chloramphenicol (30 µg), cefotaxime (30 µg), ceftazidime (30 µg), meropenem (10 µg), trimethoprim–sulphamethoxazole (1.25/23.75 µg), amoxicillin–clavulanic acid (20/10 µg), piperacillin–tazobactam (100/10 µg), ceftriaxone (30 µg), cefepime (30 µg), amikacin (30 µg) and ciprofloxacin (5 µg). These antimicrobial agents were selected based on current availability and frequently prescribed for the management of bacterial infections in Ethiopia [21].

Anti-fungal susceptibility tests for Amphotericin B (10 µg), caspofungin (5 µg), flycytosine, fluconazole (25 µg), Micafungin and Voriconazole (1 µg) were done for all yeasts isolates by automated VITEK 2 compact system (bioMérieux, France) using AST-YS01 cards. The quality controls of the drugs were checked for its performance by using *E. coli* (ATCC-25922), *S. aureus* (ATCC-25923), *P. aeruginosa* (ATCC-27853) and *C. albicans* (ATCC-10231).

### Data collection

Socio-demographic data and clinical status of patients were collected through a standard questioner.

### Statistical analysis

Data were collected analyzed using WHONET and R software programs. Simple and multiple logistic regression analyses were used to assess the possible risk factors of bloodstream infections. Multiple logistic regression models were done only for variables with P-value less than 0.25. P-value < 0.05 was considered statistically significant for all cases.

### Ethical issues

The study was conducted after obtaining ethical clearance and approval from the department of medical laboratory sciences, Addis Ababa University who had ethics committee to approve the study on humans (DREERC/452/19/MLS). An official permission letter was obtained from Addis Ababa Public health Research and Emergency Directorate for all referral hospitals for sample collection. Data collection was started after written informed consent/ascent was obtained from the study participants. Informed consent was also obtained from parent/guardian of study participants below 16 years of age.

## Results

### Socio-demographic characteristics of patients

**Fig. 1:**
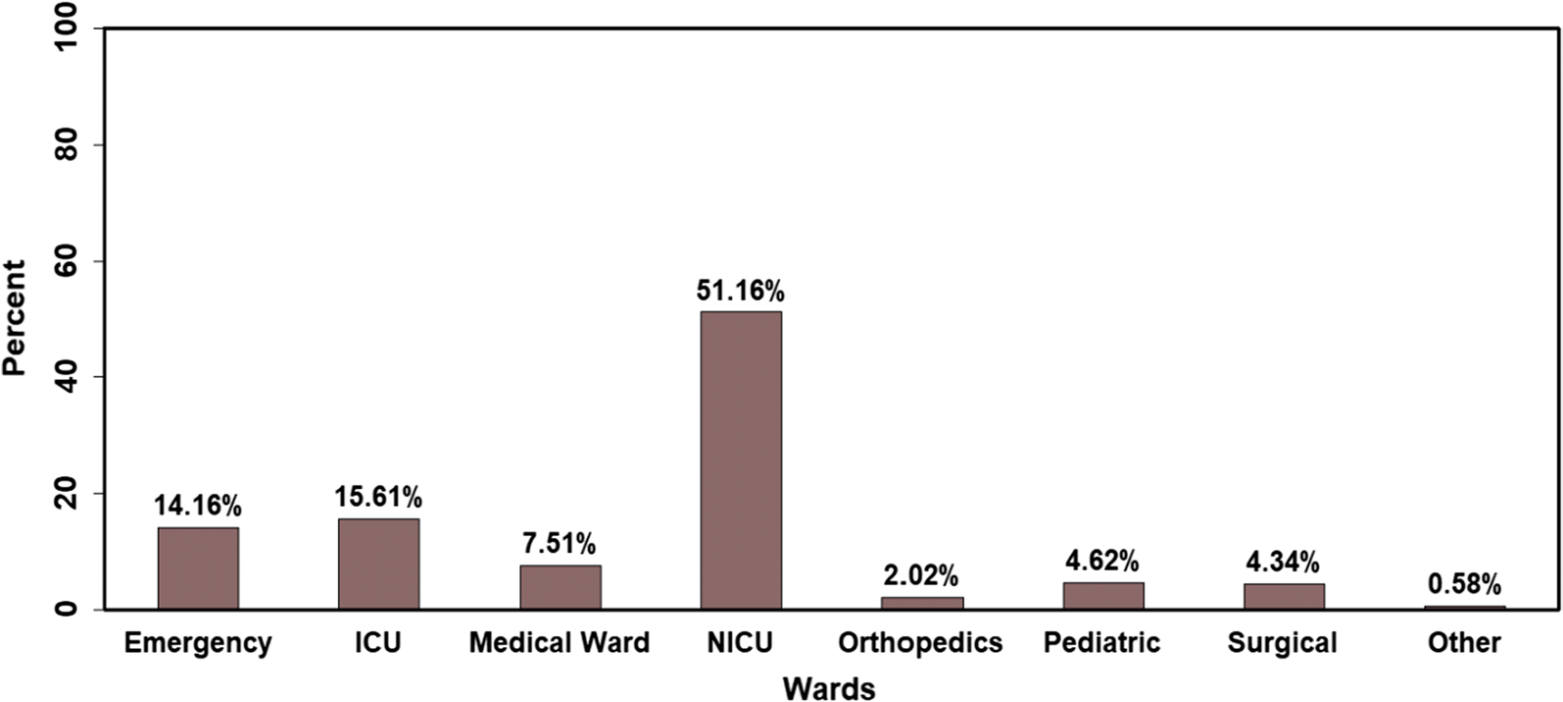
Distribution of patients with suspected BSIs referred to EPHI, Clinical Bacteriology and Mycology reference laboratory from February 23 to June 23, 2020 according to the hospital wards

The study involved a total of 346 patients with suspected BSIs. Out of them, 126 (36.42%) were female. The majority of the study participants were infants 186 (53.76%) and few participants 16 (4.62%) were elderly age groups (Table 1). More than half participants in the study were from NICU (51.16%) (Fig. 1).

**Table 1:** Demographic characteristics of study participants referred to Clinical Bacteriology and Mycology laboratory from February 23 to June 23, 2020

Variables	Category	N (%)
Gender	Female	126 (36.42)
	Male	220 (63.58)
Age	Infant (< 1yearr)	186 (53.76)
	Children (1–12 years)	27 (7.80)
	Adult (13–64 years)	117 (33.82)
	Elderly (> 65 years)	16 (4.62)
Place of residence	Outside of Addis Ababa	211 (60.98)
	Addis Ababa	135 (39.02)

### Clinical condition of study participants

Invasive medical devices such as catheter usage were observed in 178 (54.60%) study participants during their hospital stay. Patient histories revealed that they had different underlying clinical conditions. Most study participants 169 (48.84%) developed sepsis. One hundred twenty-five (36.13%) of the patients had a previous history of hospital admission related to similar symptoms with the current infections or with other health problems. Other comorbidities were also observed in study participants. These included: history of surgery 70 (20.23%), prolonged rupture of membrane 15 (4.34%), malignancy 20 (5.78%), diabetic mellitus 28 (8.09%), hepatic problem 29 (8.38%), hemodialysis 12 (3.47%) and 32 (9.25%) had other complications. One patient was with a history of bone marrow transplantation.

The majority of patients 286 (82.66%) were treated with empiric antibiotics prior to blood culture results. Of these, most of the patients (67.84%) were treated with a combination of aminoglycoside, Beta-lactam inhibitor and third generation cephalosporins. The rest of the participants (25.87%) had taken a combination of carbapnem and glycopeptide. A few patients (6.29%) were treated with metronidazole as well.

### Fungal and bacterial isolates

**Fig. 2:**
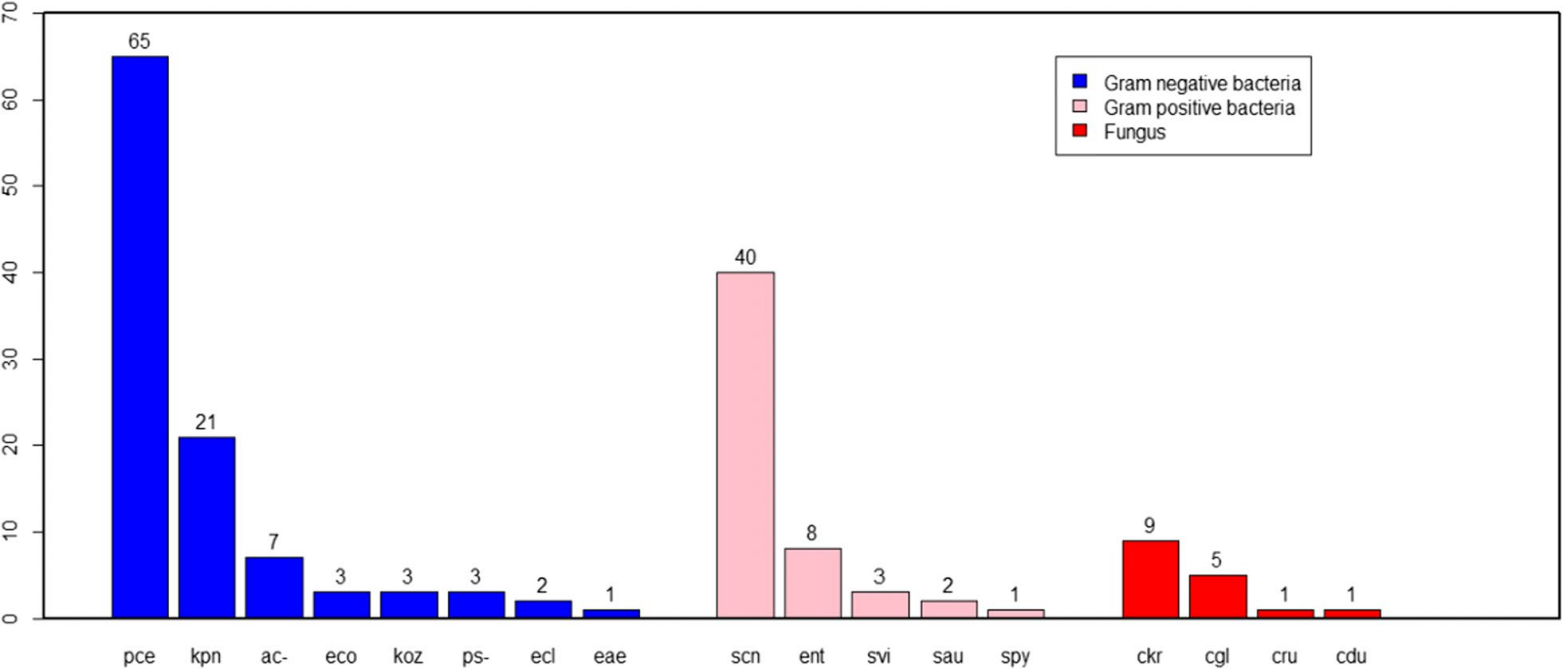
Prevalence of microbial isolates from patients referred to Clinical Bacteriology and Mycology laboratory. Pce—*Burkholderia cepacia*; kpn—*Klebsiella pneumonia*; ac—*Acinetobacter* spp.; eco—*Escherichia coli*; Koz—*Klebsiella ozaenae*; ps—*Pseudomonas* spp.; ecl—*Enterobacter cloacae*; eae—*Enterobacter aerogenes*; scn—*Coagulase negative staphylococcus*; ent—*Enterococcus* spp.; svi—*Viridans* spp.; sau—*Staphylococcus aureus*; spy—*Streptococcus pyogens*; ckr—*Candida krusei*; cgl—*Candida glabrata*; cru—*Candida rugosa*; cdu—*Candida dubliniensis*

Positive growth was observed in 175 (50.58%) isolates under aerobic cultural environments. The isolates were considered as contaminant when recovered from one bottle and excluded from our results. Only isolates recovered from both bottle were considered as true pathogen and included in analysis. All positive blood samples were detected for a single type of organism. From the recovered isolates 54 (30.86%) were Gram-positive bacteria, 105 (60%) were Gram-negative bacteria and 16 (9.14%) were fungal isolates. Among Gram-positive isolates, Coagulase-negative staphylococcus (74.07%) was the predominant bacteria followed by *Enterococcus* spp. (14.81%). On the other hand, *Burkholderia cepacia* was the predominant bacteria among Gram-negative bacterial isolates which accounted for (61.90%). All fungal isolates identified were non-*C. albicans* spp. The most frequently isolated fungi was *C. krusei* (56.25%) (Fig. 2).

### Antimicrobial resistance patterns

The microbial isolates showed varying degrees of resistance to different antibiotics tested. Overall, Gram-positive bacteria exhibited a high degree of resistance to Penicillin. Methicillin resistance defined as resistance to the antibiotic methicillin and other common antibiotics, such as amoxicillin, oxacillin, and penicillin [22]. MRSA was detected in 89.7% of Coagulase-negative staphylococcus (Table 2).

**Table 2:** Antibiotic resistance patterns of Gram-positive bacteria isolated from blood cultures of patients referred to Clinical Bacteriology and Mycology reference laboratory

Bacterial isolate	Antibiotic							
		PEN	OXA	GEN	AMP	VAN	CHL	CTX
*Coagulase negative staphylococcus*	%R	40 (100)	40 (89.7)	39 (32.5)	NT	NT	NT	NT
*Enteroccus* spp.	%R	8 (75)	NT	NT	NT	8 (0)	8 (12.5)	NT
*Viridans* spp.	%R	NT	NT	NT	NT	3 (0)	NT	3 (33.3)
*S. aureus*	%R	2 (100%)	2 (0)	2 (0)	NT	2 (0)	NT	NT

*PEN* penicillin; *OXA* oxacillin; *GEN* gentamycin; *VAN* vancomycin; *CHL* chloramphenicol; *CTX* cefotaxime; *FEP* cefepime; *NT* not tested

Antimicrobial resistance levels of Gram-negative organisms for the most commonly causing BSIs were relatively high. *K. pneumoneia* showed the highest resistance in folate pathway inhibitors (85.7%), floroquionoles (83.3%), aminoglycoside (84.2%) and third-generation cephalosporins (81%). From the member of non-enterobacterciae *Acintobacter* spp. showed extreme resistance to ciprofloxacin (100%). On the contrary, *Pseudomonas* spp. was susceptible to most of the antibiotics tested. Multidrug drug resistance was observed in 65% of Gram-negative bacterial isolates (Table 3).

**Table 3:** Antibiotic resistance patterns of Gram-negative bacteria isolated from blood cultures among patients referred to Clinical Bacteriology and Mycology laboratory

Bacterial isolate	CAZ	MEM	SXT	AMC	TZP	CRO	FEP	AMK	GEN	TOB	CIP	CHL
*B. cepacia*	65 (4.62)	65 (0)	65 (1.53)	NT	NT	NT	NT	NT	NT	NT	NT	NT
*K. pneumoniae*	21 (76.2)	21 (52.4)	21 (85.7)	20 (60)	19 (31.6)	21 (81)	21 (66.7)	21 (0)	20 (65)	21 (84.2)	6 (83.3)	21 (47.7)
*Acinetobacter* spp.	7 (57.1)	7 (71.4)	NT	NT	7 (57.2)	7 (42.9)	7 (42.9)	7 (0)	7 (71.4)	7 (71.4)	6 (100)	NT
*E. coli*	3 (33.3%)	3 (0)	3 (33.3)	3 (33.3)	3 (0)	3 (66.7)	3 (66.7)	3 (0)	3 (33.3)	2 (50)	1 (0)	3 (0)
*K. ozaenae*	3 (66.7)	3 (0)	3 (66.7)	3 (0)	3 (0)	3 (100)	3 (33.3)	3 (0)	3 (33.3)	3 (100)	1 (0)	3 (33.3)
*Pseudomonas* spp.	3 (0)	3 (0)	NT	NT	3 (0)	NT	3 (33.3)	3 (0)	3 (0)	3 (0)	2 (0)	NT
*E. cloacae*	2 (50)	2 (0)	2 (100)	2 (100)	2 (0)	2 (0)	2 (0)	2 (0)	2 (100)	2 (100)		2 (0)
*E. aerogenes*	1 (100)	1 (0)	1 (100)	NT	1 (0)	NT	NT	NT	NT	1 (0)	1 (0)	1 (100)

*CAZ* ceftazidime; *MEM* meropenem; *SXT* trimethoprim–sulphamethoxazole; *AMC* amoxicillin–clavulanic acid; *TZP* piperacillin–tazobactam; *CRO* ceftraxone; *FEP* cefipime; *AMK* amikacin; *GEN* gentamycin; *TOB* tobramycin; *CIP* ciprofloxacin; *CHL* chloramphenicol; *NT* not tested

The antimicrobial susceptibility patterns of *Candia* species were performed using Vitek compact2 machine according to the M44 guideline [23]. *C. rugosa* and *C. dubliniensis* were resistance to fluconazole (100%). All *Candida* isolates were susceptible to voriconazole (Table 4).

**Table 4:** Antifungal resistance patterns of fungal isolates from blood culture of patients referred to Clinical Bacteriology and Mycology laboratory

Fungal isolate		AMB	CAS	FCT	FLU	MIF	VOR
*C. krusei*	%R	9 (0)	9 (0)	9 (22.2)	9 (77.8)	9 (0)	9 (0)
	%S	9 (100)	9 (100)	9 (77.8)	9 (22.2)	9 (100)	9 (100)
*C. glabrata*	%R	5 (40)	5 (20)	5 (20)	5 (80)	5 (20)	5 (0)
	%S	5 (60)	5 (80)	5 (80)	5 (20)	5 (80)	5 (100)
*C. rugosa*	%R	1 (0)	1 (0)	1 (100)	1 (100)	1 (0)	1 (0)
	%S	1 (100)	1 (100)	1 (0)	1 (0)	1 (100)	1 (100)
*C. dubliniensis*	%R	1 (0)	1 (0)	1 (0)	1 (100)	1 (0)	1 (0)
	%S	1 (100)	1 (100)	1 (100)	1 (0)	1 (100)	1 (100)

*AMB* amphotericin B; *CAS* caspofungin; *FCT* flycytosine; *FLU* fluconazole; *MIF* micafungin; *VOR* voriconazole

### Multidrug resistance pattern of bacterial isolates

Multidrug drug resistance (MDR) is occurs when an organism acquired non-susceptibility to at least one agent in three or more antimicrobial categories [24]. MDR level was 65% that was only seen among Gram-negative bacterial isolates. Highest MDR was observed in *K. pneumoniae* and *Acinitobacter* spp. The *Pseudomonas* spp. and *B. cepacia* found to be non MDR for the antibiotics tested (Table 5).

**Table 5:** Multidrug resistance pattern of bacterial isolates from blood culture of patients referred to Clinical bacteriology and Mycology laboratory

Bacterial isolate	Ro	R1	R2	R3	R4	R5	R6	R7	Total MDR
*K. pneumoneia* (*N* = 21)	1 (4.8)	3 (14.3)	2 (9.5)	2 (9.5)	5 (23.8)	3 (14.3)	0 (0)	5 (23.8)	15 (71.4)
*Acinitobacter* spp. (*N* = 7)	0 (0)	0 (0)	1 (14.3)	3 (42.9)	3 (42.9)	0 (0)	0 (0)	0 (0)	6 (85.7)
*E. coli* (*N* = 3)	0 (0)	1 (33.3)	1 (33.3)	0 (0)	1 (33.3)	0 (0)	0 (0)	0 (0)	1 (33.3)
*K. ozenae* (*N = 3)*	1 (33.3)	0 (0)	1 (33.3)	0 (0)	0 (0)	1 (33.3)	0 (0)	0 (0)	1 (33.3)
*Pseudomeunos* spp. (*N* = 3)	2 (66.7)	1 (33.3)	0 (0)	0 (0)	0 (0)	0 (0)	0 (0)	0 (0)	0 (0)
*E. cloacae* (*N* = 2)	0 (0)	0 (0)	0 (0)	0 (0)	2 (100)	0 (0)	0 (0)	0 (0)	2 (100)
*E. aeroagens* (*N* = 1)	0 (0)	0 (0)	0 (0)	1 (100)	0 (0)	0 (0)	0 (0)	0 (0)	1 (100)
Total (*N* = 40)	4 (10)	5 (12.5)	5 (12.5)	6 (15)	11 (27.5)	4 (10)	0 (0)	5 (12.5)	26 (65)

*R1* resistant for 1 antibiotic, *R2* resistant for 2 antibiotics, *R3* resistant for 3 antibiotics, *R4* resistant for 4 antibiotics, *R5* resistant for 5 antibiotics, *R6* resistant for 6 antibiotics, *R7* resistant for 7 antibiotics

### Risk factors for BSIs

In this study different clinical and socio-demographic factors were observed. The association of prevalence of bacterial infections with independent variables was initially analyzed using a simple regression model. On the basis of cruds odd ratio, the candidate risk factors for the statistical model were sex, age, place of residence, history of admission, sepsis, surgery, empirical therapy, diabetic mellitus, hepatic problem, and utilization of complicated devices at 0.25 level of significant. The candidate risk factors were filtered using multiple logistic regression models. The model suggested sex (P-value = 0.007), age (P-value < 0.001) and utilization of medical devices (P-value = 0.003) were significant risk factors for bacterial infections (Table 6).

**Table 6:** Risk factor associated with bacterial cause of blood stream infections among patients referred to Clinical Bacteriology and Mycology reference laboratory

Characteristics	Categories	Bacterial infection			Pce		Kpn		Scn	
		Number (%)	AOR	P value	AOR	P value	AOR	P value	AOR	P value
Sex (ref = male)	Female	74 (58.70)	2.05	0.007	0.76	0.424	1.72	0.241	2.68	0.007
	Male	86 (39.09)								
Age (ref = adult)	Infant (< 1 year)	124 (66.67)	5.69	0.000		0.984	2.29	0.184	1.98	0.125
	Children (1–17)	5 (18.52)	0.66	0.456	1.11	0.999	1.06	0.959	0.67	0.627
	Adult (18–64)	28 (23.93)								
	Elderly (> 64)	3 (18.75)	0.62	0.484	1.04	0.999	1.61	0.682	0.57	0.617
Place of residence (ref = rural)	Urban	71 (52.59)	0.82	0.458	0.80	0.51	0.57	0.594	0.71	0.359
	Rural	89 (42.18)								
Surgery (ref = no)	Yes	25 (35.71)	0.86	0.465	1.59	0.371	1.64	0.41	0.71	0.538
Ever admitted (ref = no)	Yes	46 (36.8)	0.82	0.652	0.30	0.006	0.75	0.59	2.64	0.012
SEPSIS (ref = no)	Yes	93 (55.03)	0.97	0.899	0.70	0.304	1.07	0.888	0.94	0.878
Utilization of complicated devices (ref = no)	Yes	99 (52.66)	2.24	0.003	3.52	0.000	1.39	0.508	0.69	0.329

Pce—*Burkolderia cepacia*; Scn—Coagulase negative staphylococcus; Kpn—*Klebsiella pneumoniae*

A multiple regressions model was also done for the predominant bacteria with demographic and clinical conditions. *B. cepacia*, coagulase negative-staphylococcus and *K. pneumoniae* were the predominant bacteria associated with the independent variable. History of admission (P-value = 0.006) and utilization of medical devices (P-value < 0.001) have significant association with *B. cepacia*. On the other hand, sex (P-value = 0.007) has significant association with infection with coagulase negative staphylococcus. *K. pneumoniae* has not significant association with all demographic and clinical conditions.

## Discussion

Blood stream infections are regarded as the most devastating human diseases that lead to complex treatment procedure [16, 25]. The current study mainly illustrated the magnitude of BSIs along with the main risk factors. To our knowledge, this study was the first in addressing *B. cepacia* as a cause of BSIs in Ethiopia’s hospital setting particularly from NICU wards. Furthermore, this study differs from previous research conducted in the same laboratory in BSIs with identification and susceptibility testing for antifungal agents that was not considered a routine testing procedure in many laboratories.

In our study higher prevalence of blood culture confirmed cases [175 (50.58%)] were observed compared to studies conducted in Port Blair India 14.24% [26] and Kanpur 22.3% [27]. The differences may be due to blood specimen volume, time of blood culture taken and epidemiological variation of etiologic agents [2, 4]. Our finding might be higher due to the emerging of new nosocomial pathogen *B. cepacia*.

The percentage of bacterial isolation in this study was 159 (45.95%), which was approximately similar to the previous study conducted in Ethiopia [164 (32.8%)] [12]. In contrast to the above finding, different studies conducted in European, African countries, and Ethiopia reported a lower isolation rate of bacterial infections [13, 28–34]. The possible reason for this variation may be the predominance of *B. cepacia* detection that may increase the positivity rate in the present study.

The prevalence of bacterial blood stream infections in relation to age groups illustrated that blood culture positivity was higher among infants (77.36%) than other age groups. This finding was in line with the study performed in rural tertiary care hospital in India [25], Gonder university hospital [34] and Nigeria [13]. The predominance of bacterial BSIs among infants may be due to immaturity of the immune system which contributes to ease of susceptibility to infections [35].

The predominant pathogens causing BSIs in our study were found to be Gram-Negative bacteria, which is similar to other studies in Rim Hospital [32], Nigeria [13], Jimma [36], Addis Ababa [37] and Tikur Anbessa [31]. The higher percentage of Gram-negative bacteria in this study most likely due to a higher number of *B. cepacia* which is emerging as outbreak NICU wards.

Bacterial isolation varies among country to country. *B. cepacia*, Coagulase-negative staphylococcus, *K. pneumoneia*, *E. coli*, *Acinetobacter* spp. and *Pseudomonas* spp. were the most commonly isolated bacteria that caused bloodstream infections in this study, which is more or less similar to previous studies [13, 28, 38–40]. Among the Gram-negative bacteria *B. cepacia* was found as the highest percentage. The predominancy of this bacteria may be due to about 77.36% of patients in the current study was infants from NICU and all of these isolates were identified from these patients. *B. cepacia* is an important nosocomial pathogen that causes outbreak in hospital settings especially in neonatal units. It is also capable of adhering to various medical instruments and colonizing solutions taken as an injection for medication that may be capable of transmitting to neonates [41].

Like many other studies [16, 19, 30, 35, 42–45], Coagulase-negative staphylococcus was the predominant Gram-positive organism in our study as a causative agent of BSIs. The high prevalence of Coagulase-negative staphylococcus could be the higher number of blood samples in our study were from neonatal intensive care unit which was frequently associated with the utilization of intravascular devices that serve as portals of entry to the bloodstream [46].

Fungemia has been confirmed in 9 (9.14%) cases. The result was consistent with a study conducted in New York City 9.8% [47], Saudi Arabia 9.5% [16] and India 9.2% [48]. All fungemia infections in this study were due to non-*C. albicans* species which is in agreement with other studies [27, 43, 49]. But our finding was different from studies conducted in Turkey [50] and India [14] which found *C. albicans* as a cause of BSIs. The major risk factors leading to fungal infection in our study were prolonged hospital stay and previous broad-spectrum antimicrobial treatment [14].

*Klebsiella* spp. showed high resistance to folate pathway inhibitors, floroqunioles, aminoglycoside, and βeta lactamases inhibitors. Third-generation and fourth-generation cephalosporins also exhibited very weak activity against this organism. A similar finding was noted among studies carried out in Nepal [35], Gonder university hospital [34] and India [51].

Among non-fermenter, *Acinetobacter* spp. showed extremely resistance to fluoroquinolones and carbnem drugs. They were also moderately resistance to other antibiotics such as aminoglycoside, third and fourth generation cephalosporin, and aminoglycoside and βeta lactam inhibitors. This finding was consistent with the study done in Iran which showed that *Acinetobacter* spp. was the most resistant bacteria against most antibiotics tested [52]. The main reason for this problem may be inappropriate use of antibiotics and lack of standard antibiotic policy in the hospital. Amikiacin was the most effective antibiotic against Gram-negative agents that is comparable in studies performed in Asian and Arab countries [49, 52–54].

In our study, a higher degree of resistance was observed in penicillin among Gram-positive organisms which showed conformity with studies done in Jimma [33] and Afghanistan [44]. All Gram-positive bacteria were sensitive to vancomycin that is similar to other studies [19, 43, 51, 53, 55]. However, different from the study in India, vancomycin resistance and intermediate *S. aureus* were observed [48]. The difference may be the number of isolates that were identified. MRSA was observed in the predominant organism Coagulase-negative staphylococcus which similar to study in tertiary care hospital in India [51] and Nepal [35].

In our study, the highest resistance rate of azole drugs observed in fluconazole in *C. krusei* showed similarity in studies conducted in India [38] and Turkey [50]. However, Study in Qatar showed most *Candida* spp., were sensitive to fluconazole, which is different from our study [49]. There is no resistance against voriconazole in all candida isolates which was in line with study done in Turkey [50].

Many factors contribute to the prevalence of BSIs. In the current study Sex, Age, and utilization of complicated devices were independent risk factors for BSIs caused by bacterial infection. This finding agrees with a study conducted in Gonder university hospital that has a significant association between age and BSIs [34].

## Conclusions

In this study, the frequency of Gram-negative bacteria that causes BSIs was higher compared to Gram-positive bacterial and fungal isolates. Fungemia due to non-*C. albicans* was common fungal causative agent. Sex, age, and utilization of complicated devices were found to be the main risk factor that causes BSIs associated with bacterial infections. Both Gram-positive and Gram-negative bacteria showed an increasing level of resistance to most antibiotics that have been used for empirical therapy. This highlights continuing investigation of etiologic agents and assessment of infection control is necessary to reduce the disease impact. In addition, it is important to revise treatment guidelines based on the predominant pathogen and resistance patterns at the health facility level.

## Limitation of the study

All specimens during the study period were collected from Addis Ababa health care facilities that could not represent the national data of Ethiopia. There was no information whether the cases were community-acquired or healthcare-acquired infections, even though the majority of the isolates were identified from NICU.

## Data Availability

The datasets used and/or analyzed during the current study are available from the corresponding author on reasonable request.
